# Estimation of genetic variance for macro- and micro-environmental sensitivity using double hierarchical generalized linear models

**DOI:** 10.1186/1297-9686-45-23

**Published:** 2013-07-04

**Authors:** Han A Mulder, Lars Rönnegård, W Freddy Fikse, Roel F Veerkamp, Erling Strandberg

**Affiliations:** 1 Wageningen UR Livestock Research, Animal Breeding and Genomics Centre, PO Box 65, 8200 AB, Lelystad, The Netherlands; 2 Animal Breeding and Genomics Centre, Wageningen University, PO Box 338, 6700 AH, Wageningen, The Netherlands; 3 School of Technology and Business Studies, Högskolan Dalarna SE-79188, Falun, Sweden; 4 Department of Animal Breeding and Genetics, Swedish University of Agricultural Sciences, Box 7023, SE-75007, Uppsala, Sweden

## Abstract

**Background:**

Genetic variation for environmental sensitivity indicates that animals are genetically different in their response to environmental factors. Environmental factors are either identifiable (e.g. temperature) and called macro-environmental or unknown and called micro-environmental. The objectives of this study were to develop a statistical method to estimate genetic parameters for macro- and micro-environmental sensitivities simultaneously, to investigate bias and precision of resulting estimates of genetic parameters and to develop and evaluate use of Akaike’s information criterion using h-likelihood to select the best fitting model.

**Methods:**

We assumed that genetic variation in macro- and micro-environmental sensitivities is expressed as genetic variance in the slope of a linear reaction norm and environmental variance, respectively. A reaction norm model to estimate genetic variance for macro-environmental sensitivity was combined with a structural model for residual variance to estimate genetic variance for micro-environmental sensitivity using a double hierarchical generalized linear model in ASReml. Akaike’s information criterion was constructed as model selection criterion using approximated h-likelihood. Populations of sires with large half-sib offspring groups were simulated to investigate bias and precision of estimated genetic parameters.

**Results:**

Designs with 100 sires, each with at least 100 offspring, are required to have standard deviations of estimated variances lower than 50% of the true value. When the number of offspring increased, standard deviations of estimates across replicates decreased substantially, especially for genetic variances of macro- and micro-environmental sensitivities. Standard deviations of estimated genetic correlations across replicates were quite large (between 0.1 and 0.4), especially when sires had few offspring. Practically, no bias was observed for estimates of any of the parameters. Using Akaike’s information criterion the true genetic model was selected as the best statistical model in at least 90% of 100 replicates when the number of offspring per sire was 100. Application of the model to lactation milk yield in dairy cattle showed that genetic variance for micro- and macro-environmental sensitivities existed.

**Conclusion:**

The algorithm and model selection criterion presented here can contribute to better understand genetic control of macro- and micro-environmental sensitivities. Designs or datasets should have at least 100 sires each with 100 offspring.

## Background

The term “genotype by environment (G × E) interaction” refers to the fact that the best genotype in one environment may not be the best genotype in another environment [1] and that genotypes differ in their response to environmental factors, which means that genetic variance for environmental sensitivity or phenotypic plasticity exists [2]. Some environmental factors (e.g., temperature, soil, diet, etc.) are identifiable and can be categorised (e.g., temperate or tropical climate) or quantified (e.g., temperature) and thus are referred to as macro-environmental factors. Other environmental factors are unknown and referred to as micro-environmental factors [1]. Therefore, genetic variance in macro-environmental sensitivity is the genetic variance due to known environmental factors and can be expressed as the genetic variance in the slope of a reaction norm when environments can be quantified on a continuous scale. If environments are categorized, then phenotypes in different environments are considered as separate traits and the genetic covariances between environments are a measure of genetic variation in macro-environmental sensitivity. Genetic variance in micro-environmental sensitivity is the genetic variance due to unknown environmental factors and can be expressed as differences in environmental variance, sometimes called genetic heterogeneity of environmental variance [3].

Numerous studies in the last 70 years have studied G × E interactions or genetics of macro-environmental sensitivity in animal and plant breeding as well as in evolutionary biology [4-6]. Different modeling approaches have been used [4]. In recent years, reaction norm models have been applied in animal breeding to better understand the environmental factors that determine G × E interactions [7-9]. In evolutionary genetics, many experiments on Drosophila and other laboratory species have been carried out to understand the genetics of macro-environmental sensitivity or phenotypic plasticity [6,10,11]. Other examples of studies on wild life populations include analyses of great tit [12] and butterfly [13] populations that showed the existence of genetic variation in phenotypic plasticity to temperature change.

The genetics of micro-environmental sensitivity or environmental variance have been studied less extensively than the genetics of macro-environmental sensitivity. In evolutionary genetics, several studies have focused on canalization, i.e. selection for reduced variance [14-16]. Recently, there has been renewed interest on this topic due to the development of methods to estimate genetic variance in environmental variance, e.g. Bayesian methods [17,18] and double hierarchical generalized linear models (DHGLM) in a REML setting [19]. Hill and Mulder [20] reviewed 14 studies on this subject and concluded that there is empirical evidence for the existence of genetic variance in environmental variance.

Although there is substantial evidence for genetic variance in macro- and micro-environmental sensitivities, very few studies have studied them together or studied their genetic relationship. Jinks and Pooni [21] postulated the concepts of macro- and micro-environmental sensitivity and studied both in *Nicotiana rustica* but did not investigate the relationships between these two types of environmental sensitivity. However, Perkins and Jinks [22] reported that both types of environmental sensitivity were weakly genetically correlated for most traits in *Nicotiana rustica*. In a study on *Populus*, Wu [23] showed that micro-environmental sensitivity was less heritable than macro-environmental sensitivity and that they were weakly genetically correlated. In another paper, also on *Populus*, Wu and O'Malley [24] detected different sets of genes for macro- and micro-environmental sensitivities. In a mapping study of QTL for macro- and micro-environmental sensitivities in barley, Kraakman et al. [25] identified only a few QTL for micro-environmental sensitivity, but did not detect any QTL for macro-environmental sensitivity. Yampolsky and Scheiner [26] observed a weak genetic correlation between developmental noise, similar to micro-environmental sensitivity, and macro-environmental sensitivity in *Daphnia Magna*. In *Drosophila melanogaster*, both positive and negative genetic correlations between macro- and micro-environmental sensitivities have been reported [27,28]. In farm animals, the genetic correlation between macro- and micro-environmental sensitivities has not been investigated. In addition, to date, no suitable method was available to estimate such correlations in outbred populations and the requirements in terms of number of families and family sizes of the designs necessary to estimate genetic parameters of macro- and micro-environmental sensitivities were unknown.

The objectives of this study were to extend the double hierarchical generalized linear model (DHGLM) of Rönnegård et al. [19] with a reaction norm model to estimate genetic variance for macro- and micro-environmental sensitivities and to investigate bias and precision of estimated variance components in designs resembling dairy cattle populations. In addition, we evaluated Akaike’s information criterion (AIC) using approximated h-likelihood to select the best fitting model and studied situations in which true genetic and best fitting statistical models differed. Finally, we applied the model to lactation milk yield in dairy cattle.

## Methods

In this section, we describe the assumed quantitative genetic model underlying genetic variance for macro- and micro-environmental sensitivities, the statistical model to estimate genetic variance in macro- and micro-environmental sensitivities, and the AIC based on an approximation of the h-likelihood that can be used to select the best fitting model. Finally, we present the simulation used to test the statistical model and describe the evaluated scenarios as well as an application to milk yield in dairy cattle.

### Quantitative genetic model

Here, we assume that genetic variance for macro-environmental sensitivity is expressed as the genetic variation in the slope of a linear reaction norm. Genetic variance in micro-environmental sensitivity implies that genotypes respond differently to one or several unknown environmental factors and thus, we assume that it is expressed as genetic variance in environmental variance according to an exponential model [29]. Thus, the quantitative genetic model underlying genetic variance in macro- and micro-environmental sensitivities can be formulated as:

(1) $$\begin{array}{l} P\;=\;\mu \;+\;A_{\mathrm{int}}\;+\;A_{\mathrm{sl}}x\;\\ \;+\exp\left(0.5\ln\left(\sigma_{E}^{2}\right)\;+\;0.5A_{v}\right)\epsilon \end{array}$$

where *P* is the phenotype, *μ* is the population mean for the phenotype, *x* is the environmental parameter (continuous or discrete) that cause genotypes to respond differently*, A*_*int*_*, A*_*sl*_ and *A*_*v*_ are the additive genetic or breeding values for the intercept, for the slope of the reaction norm (= macro-environmental sensitivity), and for the environmental variance (= micro-environmental sensitivity), respectively, $\sigma_{E}^{2}$ is the environmental variance of the exponential model and *ϵ* is a scaled environmental deviation with variance one. Note that the average environmental variance is $\bar{\sigma_{E}^{2}}=\sigma_{E}^{2}\exp\left(0.5\sigma_{A_{v}}^{2}\right)$[3]. The additive genetic values *A*_*int*_*, A*_*sl*_ and *A*_*v*_ have variance

$$G=\left[\begin{array}{ccc} \sigma_{A_{\mathrm{int}}}^{2} & \sigma_{A_{\mathrm{int}},A_{\mathrm{sl}}} & \sigma_{A_{\mathrm{int}},A_{v}}\\ & \sigma_{A_{\mathrm{sl}}}^{2} & \sigma_{A_{\mathrm{sl}},A_{v}}\\ & & \sigma_{A_{v}}^{2} \end{array}\right]$$

### Statistical model

The basis of the statistical model is the DHLGM as presented by Rönnegård et al. [19]. The original DHGLM algorithm in Rönnegård et al. [19] iterates between a linear mixed model for the phenotypic observations: *y*_*i*_ = *P*_*i*_ = phenotypic observation of animal *i*; and a Gamma GLM for the residual variance *φ*_*i*_, where $\;\varphi_{i}\;=\;E\left(\frac{{\hat{e}}_{i}^{2}}{1-h_{i}}\right),\;{\hat{e}}_{i}^{2}$ is the squared estimated residual of *y*_*i*_ and *h*_*i*_ is the diagonal element of the hat-matrix of **y** corresponding to *y*_*i*_[30]. Felleki et al. [31] extended the model of [19] by allowing estimation of the genetic covariance ( $\sigma_{a,a_{v}}$) between the genetic effect in the mean model and the genetic effect in the variance model (Equation (2)) in the absence of a linear reaction norm model and reported on computational details and a theoretical assessment of this algorithm. In brief, equivalent to using a Gamma GLM, linearizing $\frac{{\hat{e}}_{i}^{2}}{1-h_{i}}$ around its current fitted value results in vector **ψ** and yields the following bivariate linear model:

(2) $$\left[\begin{array}{c} y\\ \psi \end{array}\right]=\left[\begin{array}{cc} X & 0\\ 0 & X_{v} \end{array}\right]\left[\begin{array}{c} b\\ b_{v} \end{array}\right]+\left[\begin{array}{cc} Z & 0\\ 0 & Z_{v} \end{array}\right]\left[\begin{array}{c} a\\ a_{v} \end{array}\right]+\left[\begin{array}{c} e\\ e_{v} \end{array}\right]$$

where **X(X**_**v**_**)** is the incidence matrix for fixed effects for **y** (**ψ**), **b (b**_**v**_**)** is the vector with solutions for fixed effects for **y**(**ψ**), **Z(Z**_**v**_**)** is the incidence matrix for relating observations of **y**(**ψ**) to the additive genetic values **a (a**_**v**_**)** for phenotype (environmental variance) $\left[\begin{array}{c} a\\ a_{v} \end{array}\right]\sim N\left(0,\left[\begin{array}{cc} \sigma_{a}^{2} & \sigma_{a,a_{v}}\\ \sigma_{a,a_{v}} & \sigma_{a_{v}}^{2} \end{array}\right]⊗A\right)$, where **A** is the additive genetic or numerator relationship matrix and $\sigma_{a}^{2}\;\left(\sigma_{a_{v}}^{2}\right)$ is the genetic variance of **a**(**a**_**v**_). The residuals of **y** (**e**) and **ψ** (**e**_**v**_) are assumed to be independent normally distributed $\left[\begin{array}{c} e\\ e_{v} \end{array}\right]\sim N\left(\begin{array}{c} 0\\ 0 \end{array},\left[\begin{array}{cc} W^{-1}\sigma_{\in }^{2} & 0\\ 0 & W_{v}^{-1}\sigma_{\in_{v}}^{2} \end{array}\right]\;\right)$, $W=\mathrm{diag}{\left(\hat{\psi }\right)}^{-1}$ and $W_{v}=\mathrm{diag}\left(\frac{1-h}{2}\right)$**,**$\sigma_{\varepsilon }^{2}$ and $\sigma_{\varepsilon_{v}}^{2}$ are scaling variances, which are expected to be equal to 1, because **W** and **W**_**v**_ already contain the reciprocals of the residual variances per observation. Estimating $\sigma_{\varepsilon }^{2}$ and $\sigma_{\varepsilon_{v}}^{2}$ allows more flexibility. An iterative estimation procedure is required to obtain estimates for all parameters because of the dependence of the model for **y** on the results of the model for **ψ** and vice versa. The vector **ψ** and the diagonals of **W** and **W**_**v**_ are updated at each iteration until convergence. The model in Equation (2) can be considered as combining the DHGLM method [19] with the iterative method of Mulder et al. [32].

In order to consider macro- and micro-environmental sensitivity simultaneously, the model for phenotype **y** in Equation (2) was extended with a linear reaction norm model to estimate genetic variance in macro-environmental sensitivity, whereas the residual variance model using **ψ** estimates the genetic variance in micro-environmental sensitivity or environmental variance. Instead of using an animal model, we used a sire model because implementation of the animal model DHGLM resulted in severely biased estimates of variance components with single observations per animal. As an indication of poor model fit, implementation of the animal model DHGLM gave a lower adjusted profile h-likelihood (see next section “Model selection”) than an animal model without heterogeneity of residual variance. Sire models are commonly used in combination with reaction norm models [7,8,33]. In the reaction norm model, we assumed that the environmental parameter *x* (see Equation (1)) is known without error and does not need to be estimated from the data. The resulting combined macro–micro environmental sensitivity sire model can be formulated as:

(3) $$\begin{array}{l} \left[\begin{array}{c} y\\ \psi_{s} \end{array}\right]=\left[\begin{array}{cc} X & 0\\ 0 & X_{v} \end{array}\right]\left[\begin{array}{c} b\\ b_{v} \end{array}\right]+\left[\begin{array}{ccc} Z_{s} & Z_{x} & 0\\ 0 & 0 & Z_{\mathrm{sv}} \end{array}\right]\\ \;\times \left[\begin{array}{c} \begin{array}{c} s_{\mathrm{int}}\\ s_{\mathrm{sl}} \end{array}\\ s_{v} \end{array}\right]+\left[\begin{array}{c} e_{s}\\ e_{\mathrm{sv}} \end{array}\right] \end{array}$$

where $\;\psi_{s}$ is a vector with linearized values of transformed squared residuals (see calculation in Appendix), **Z**_**s**_ and **Z**_**sv**_ are respectively the incidence matrices for the sire effects for the intercept of the reaction norm and for the environmental variance, **Z**_**x**_ is the matrix with the environmental parameter *x* as a covariate for the sire effects for the slope of the reaction norm, and **s**_**int**_, **s**_**sl**_ and **s**_**v**_ are the vectors with the estimated sire effects for intercept, slope, and environmental variance. The sire effects **s**_**int**_, **s**_**sl**_, and **s**_**v**_ are assumed trivariate normally distributed $N\left(0,\frac{1}{4}G⊗A\right)$, assuming that sire (co)variances are a quarter of the additive genetic variance. The residuals of **y (e**_**s**_**)** and **ψ**_**s**_(**e**_**sv**_) are assumed to be independent normally distributed, because cov(*e*, *e*^2^) = 0, when *e* is normally distributed ( $\left[\begin{array}{c} e_{s}\\ e_{\mathrm{sv}} \end{array}\right]\sim N\left(\begin{array}{c} 0\\ 0 \end{array},\left[\begin{array}{cc} W_{s}^{-1}\sigma_{\in_{s}}^{2} & 0\\ 0 & W_{\mathrm{sv}}^{-1}\sigma_{\in_{\mathrm{sv}}}^{2} \end{array}\right]\;\right)$), where $\sigma_{\in_{s}}^{2}$ and $\sigma_{\in_{\mathrm{sv}}}^{2}$ are scaling variances for the residual variances in a sire model. Computations of **ψ**_**s**_ and of the diagonals of **W**_**s**_ and **W**_**sv**_ are different due to the use of a sire model compared to an animal model and are explained in the Appendix. The algorithm was implemented by iterating over several runs of ASReml [34]. In each ASReml run, REML-estimates of the variance components were obtained for the current values of **ψ**_**s**_, **W**_**s**_ and **W**_**sv**_. The vector **ψ**_**s**_ and the diagonals of **W**_**s**_ and **W**_**sv**_ were updated after each ASReml run. On simulated data, 10 ASReml runs were sufficient to obtain converged parameter estimates. ASReml was run with an option to check whether the variance-covariance matrices were positive definite and were forced to be positive definite [34] if they were not.

### Model selection

When working with real data, the true genetic model is not known, thus statistical inference can be used to find the statistical model that fits the data best. The h-likelihood concept can be applied when using DHGLM [35], in which the adjusted profile h-likelihood (APHL) is used to assess the significance of variance components. Because we use an iterative reweighted least square approximation in ASReml, the APHL can be approximated from the log REML likelihood (*logL*) for the bivariate model in Equation (3) fitted in ASReml, after correcting for the fact that the squared estimated residuals of **y** are used to compute the response variable **ψ**_**s**_ (see Appendix for derivation):

(4) $$\mathrm{APHL}=-2\mathrm{logL}-\sum w_{sv_{i}}{\left({\hat{\sigma }}_{\in_{\mathrm{sv}}}^{2}\right)}^{-1}-\sum \ln\left({\hat{\sigma }}_{\in_{\mathrm{sv}}}^{2}\right)/w_{sv_{i}},$$

where $w_{sv_{i}}$ is the weight for the variance model for observation *i*, i.e. the *i*^*th*^ diagonal of **W**_**sv**_. Note that Equation (4) can also be used for animal models by replacing elements $w_{sv_{i}}$ and $\sigma_{\varepsilon_{\mathrm{sv}}}^{2}$ with the corresponding elements of Equation (2). To compare nested and non-nested models, we propose to use APHL in combination with Akaike’s information criterion (AIC):

(5) AIC=APHL+2t,

where t is the number of variance parameters.

### Simulation

Monte Carlo simulation was used to evaluate the bias and precision of the estimated genetic parameters of the model and proposed algorithm. Populations of paternal half-sib families were simulated, resembling a simplified population structure in an animal breeding context with large half-sib offspring groups per sire like in dairy cattle. Either the number of sires (50, 100 or 200) was varied while keeping the number of offspring per sire at 100, or the number of offspring per sire (20, 50, 100 or 200) was varied while keeping the number of sires at 100. Sires and dams were assumed unrelated and were not selected. The offspring had phenotypes but the dams or sires did not. Phenotypes were generated for offspring according to the quantitative genetic model in Equation (1). Dams were randomly assigned to herds (herd size = 100 cows) and their offspring (daughters in the case of dairy cattle) were in the same herd. For each herd, the environmental covariate *x* was sampled from N(0,1). No fixed effects other than a general mean were simulated (*μ* = 0) but offspring were randomly allocated to contemporary groups with size 10 to investigate the effect of fixed effects on the precision of genetic parameters. Additive genetic effects were sampled from N(**0**, **G** ⊗ **A**). Different values for genetic variances and genetic correlations were used for simulation. Table 1 gives an overview of the parameter values used in the simulation, both the default and alternative values. In each scenario, only one parameter was varied at a time, i.e. other parameters were kept at their default values. For each set of parameters, 100 replicates were generated and means and standard deviations of estimates were calculated across replicates.

**Table 1:** Default and alternative parameters values used in Monte Carlo simulation

**Parameter**	**Default**	**Alternative values**
$\sigma_{A_{\mathrm{int}}}^{2}$	0.3	0.1, 0.5
$\sigma_{A_{\mathrm{sl}}}^{2}$	0.05	0.025, 0.10
$\sigma_{A_{v}}^{2}$	0.1	0.05, 0.2
Genetic correlations (see text below Equation (1))	0	0.5
Number of offspring per sire	100	20, 50, 200
Number of sires	100	50, 200
Number of replicates	100	

$\sigma_{A_{\mathrm{int}}}^{2}$ is the additive genetic variance for the intercept of the reaction norm; $\sigma_{A_{\mathrm{sl}}}^{2}$ is the additive genetic variance for the slope of the reaction norm or macro-environmental sensitivity and $\sigma_{A_{v}}^{2}$ is the additive genetic variance for micro-environmental sensitivity or environmental variance.

### Scenarios in which true genetic and statistical models differ

First, we tested situations in which the true genetic and statistical models contained both types of environmental sensitivities for different sets of genetic parameters and designs. In addition, we investigated the power of AIC to select the correct model given the true genetic model. Three situations were simulated with default parameters: presence of genetic variance in (i) macro- or (ii) micro-environmental sensitivity or in (iii) both. Four statistical models were applied to these simulated situations: a combined macro–micro environmental sensitivity model “macro–micro”, a macro-environmental sensitivity model “macro”, a micro-environmental sensitivity model “micro” and a simple model “simple” with only one additive genetic effect for the phenotype. The “macro–micro” model and the “micro” model were run for 10 ASReml runs to update the weights and the transformed squared residuals, whereas the “macro” model and the “simple” model required only one ASReml run. AIC (Equation 5) was used to select the best fitting model.

Finally, we tested scenarios in which the statistical model deviated from the true genetic model used for simulation, i.e. when model selection failed to select the right model (Table 2). These scenarios were analyzed to explore whether genetic parameters are biased when the statistical model deviates from the true genetic model and whether macro-environmental sensitivity can be detected with a micro-environmental sensitivity model or vice versa. These scenarios were run with the default set of parameters as listed in Table 1 but with various values of $\sigma_{A_{\mathrm{sl}}}^{2}$ and $\sigma_{A_{v}}^{2}$. Scenarios for which the true genetic and statistical models were the same and included either macro or micro-environmental sensitivity were not analyzed since these have been previously investigated, i.e. see [36] for macro-environmental sensitivity and [19] for micro-environmental sensitivity.

**Table 2:** Scenarios with different combinations of true genetic models and statistical models

	**Statistical model**		
**True genetic model**	**Macro ES**	**Micro ES**	**Macro and micro ES**
Macro ES	Not addressed	A	B
Micro ES	C	Not addressed	D
Macro and micro ES	E	F	G (Default in this study)

*Macro ES* = macro-environmental sensitivity; *micro ES* = micro-environmental sensitivity.

### Application to milk yield in dairy cattle

The “macro–micro”, “macro”, “micro” and “simple” models were applied to 305-day lactation milk yield of 142 565 first parity Swedish Holstein cows. Lactation yields were calculated based on test-day data, as described in [37] using the test-day interval method [38]. Seasons were defined as three-month periods (January-March, April-June, July-September, October-December). Herd-year-season (HYS) classes with less than five cows were excluded. Data on a total of 762 sires with on average 187 daughters were available and 213 of these sires had more than 100 daughters. In the model for phenotype (**y**), HYS was a fixed effect and in the model for residual variance (**ψ**_**s**_), HYS was a random effect to allow regression to the mean with few observations per HYS and avoid extreme HYS estimates. The model for **y** included a fixed fourth order polynomial for age at calving and a fixed ninth-order polynomial for lactation length, while the model for **ψ**_**s**_ included a third and a sixth order polynomial for age at calving and lactation length, respectively. The orders of polynomials were determined using a Wald test in univariate models for **y** and **ψ**_**s**_ and were kept the same for “macro–micro”, “macro”, “micro” and “simple” models. Herd-year average milk yield standardized to a mean of zero and a variance of one was used as an environmental parameter for macro-environmental sensitivity. The “macro–micro”, “macro”, “micro” and “simple” models were all run for 50 ASReml runs. After 50 runs, the change in variance components was less than 0.2% of the change between consecutive runs and therefore parameters were considered converged.

## Results

### Variance components and effect of fixed effects

Estimated genetic variances were close to their true values when simulated genetic correlations were zero, although occasionally the average value deviated slightly from the true value (Table 3). The average of the estimated genetic variance in slope $\left(={\hat{\sigma }}_{A_{\mathrm{sl}}}^{2}\right)$ was biased upwards in all cases, but the bias was small (between +2 and +13%). Surprisingly, standard deviations of ${\hat{\sigma }}_{A_{\mathrm{sl}}}^{2}$ relative to $\sigma_{A_{\mathrm{sl}}}^{2}$ were small (between 21 and 30% of $\sigma_{A_{\mathrm{sl}}}^{2}$). Note that standard deviations across replicates represent standard errors of estimates from a single replicate, e.g. an analysis of one data set. The average of the estimated genetic variance in environmental variance $\left(={\hat{\sigma }}_{A_{v}}^{2}\right)$ had on average larger deviations from the true value but no direction in bias could be detected. Standard deviations of ${\hat{\sigma }}_{A_{v}}^{2}$ relative to $\sigma_{A_{v}}^{2}$ were large (between 31 and 74%), indicating that this parameter had a low precision. In the presence of fixed effects (i.e. contemporary group) for the mean and residual variance, standard deviations of estimated parameters increased on average by 9% and the number of replicates for which the genetic variance-covariance structure was forced to be positive definite also increased. Thus, the conclusion is that estimates of genetic variances are practically unbiased but that the precision of ${\hat{\sigma }}_{A_{v}}^{2}$ is low.

**Table 3:** Means and standard deviations of estimated genetic parameters across 100 replicates when genetic correlations are zero

**Fixed effects**^**1**^	**True parameters**			**Estimated parameters (SD)**						**Np**
	$\sigma_{A_{\mathrm{int}}}^{2}$	$\sigma_{A_{\mathrm{sl}}}^{2}$	$\sigma_{A_{v}}^{2}$	$\sigma_{A_{\mathrm{int}}}^{2}$		$\sigma_{A_{\mathrm{sl}}}^{2}$		$\sigma_{A_{v}}^{2}$		
No	0.1	0.05	0.1	0.101	(0.020)	0.051	(0.012)	0.095	(0.035)	0
No	0.3	0.05	0.1	0.315	(0.053)	0.054	(0.015)	0.107	(0.046)	0
No	0.5	0.05	0.1	0.507	(0.066)	0.052	(0.013)	0.115	(0.061)	3
No	0.3	0.05	0.05	0.297	(0.047)	0.053	(0.012)	0.053	(0.029)	4
No	0.3	0.05	0.2	0.308	(0.049)	0.053	(0.014)	0.186	(0.065)	0
No	0.3	0.025	0.1	0.298	(0.047)	0.026	(0.008)	0.097	(0.041)	0
No	0.3	0.1	0.1	0.296	(0.052)	0.104	(0.021)	0.083	(0.045)	1
Yes	0.1	0.05	0.1	0.099	(0.022)	0.053	(0.014)	0.091	(0.037)	1
Yes	0.3	0.05	0.1	0.309	(0.044)	0.057	(0.015)	0.104	(0.051)	0
Yes	0.5	0.05	0.1	0.503	(0.084)	0.052	(0.014)	0.104	(0.074)	10

^1^Models have either only a mean as fixed effect (no fixed effects) or have contemporary groups as fixed effects (yes); $\sigma_{A_{\mathrm{int}}}^{2}$ = additive genetic variance of breeding value for the intercept; $\sigma_{A_{\mathrm{sl}}}^{2}$ = additive genetic variance of breeding value for the slope (= macro-environmental sensitivity); $\sigma_{A_{v}}^{2}\;$ = additive genetic variance for environmental variance (= micro-environmental sensitivity); Np = number of replicates with covariance structures forced to be positive definite.

### Effect of genetic correlations

In general, estimates of genetic variances remained close to their true values when genetic correlations were varied (<10% difference from the true value) (Table 4). When all genetic correlations were equal to 0.5, the genetic variance of environmental variance was slightly underestimated (−11%). Estimates of genetic correlations were unbiased but had very high standard deviations, especially the genetic correlation between the breeding value for slope and environmental variance ( $\rho_{A_{\mathrm{sl}},A_{v}}$). The number of replicates in which the variance-covariance structure was forced to be positive definite was in general small but slightly higher when all genetic correlations were equal to 0.5. In addition to the results shown, we simulated an extreme negative genetic correlation ( $\rho_{A_{\mathrm{sl}},A_{v}}=–0.9$), as well as an extreme positive genetic correlation ( $\rho_{A_{\mathrm{sl}},A_{v}}=\;0.9$). In these scenarios, for 40 to 60% of the replicates, the variance-covariance matrix was bended to be positive definite. Due to the large proportion of replicates with bended variance-covariance matrices, the average genetic correlation was biased towards zero. Nevertheless, the general conclusion is that estimates of genetic correlations are largely unbiased but are estimated with low precision.

**Table 4:** Means and standard deviations across 100 replicates of estimated genetic parameters when genetic correlations are not zero

**True parameters**			**Estimated parameters (SD)**						
$\rho_{A_{\mathrm{int}},A_{v}}$	$\rho_{A_{\mathrm{int}},A_{\mathrm{sl}}}$	$\rho_{A_{\mathrm{sl}},A_{v}}$	$\sigma_{A_{\mathrm{int}}}^{2}$	$\sigma_{A_{\mathrm{sl}}}^{2}$	$\sigma_{A_{v}}^{2}$	$\rho_{A_{\mathrm{int}},A_{v}}$	$\rho_{A_{\mathrm{int}},A_{\mathrm{sl}}}$	$\rho_{A_{\mathrm{sl}},A_{v}}$	**Np**
0	0	0	0.315	0.054	0.107	−0.005	0.004	0.012	0
			(0.053)	(0.015)	(0.046)	(0.165)	(0.155)	(0.249)	
0.5	0	0	0.303	0.051	0.099	0.554	−0.028	−0.007	1
			(0.047)	(0.012)	(0.048)	(0.155)	(0.136)	(0.203)	
0	0.5	0	0.303	0.053	0.094	−0.010	0.508	−0.014	1
			(0.056)	(0.012)	(0.045)	(0.200)	(0.132)	(0.239)	
0	0	0.5	0.293	0.052	0.092	0.014	0.021	0.558	2
			(0.045)	(0.013)	(0.034)	(0.185)	(0.147)	(0.208)	
0.5	0.5	0.5	0.301	0.053	0.089	0.537	0.517	0.530	6
			(0.051)	(0.013)	(0.036)	(0.171)	(0.138)	(0.192)	

$\rho_{A_{\mathrm{int}},A_{v}}$ = genetic correlation between additive genetic effects for intercept and environmental variance; $\rho_{A_{\mathrm{int}},A_{\mathrm{sl}}}$ = genetic correlation between additive genetic effects for intercept and slope; $\rho_{A_{\mathrm{sl}},A_{v}}\;$ = genetic correlation between additive genetic effects for slope (macro-environmental sensitivity) and environmental variance (micro-environmental sensitivity); $\sigma_{A_{\mathrm{int}}}^{2}$ = additive genetic variance of breeding value for intercept (true value = 0.3); $\sigma_{A_{\mathrm{sl}}}^{2}$ = additive genetic variance of breeding value for slope (= macro-environmental sensitivity; true value = 0.05); $\sigma_{A_{v}}^{2}\;$ = additive genetic variance for environmental variance (= micro-environmental sensitivity; true value = 0.10); Np = number of replicates with covariance structures forced to be positive definite.

### Effect of different designs

When varying the number of offspring per sire or the number of sire families, means of genetic variances were close to their true simulated values in most cases, except when the number of offspring per sire was 20 for which estimates for $\sigma_{A_{\mathrm{sl}}}^{2}$ and $\sigma_{A_{v}}^{2}$ were biased upwards (25% and 20%, respectively) (Table 5). In this case, 51% of the replicates had variance-covariance matrices that were forced to be positive definite, indicating that this is not a suitable design to estimate genetic parameters for macro- and micro-environmental sensitivities. When increasing the number of offspring per sire, the standard deviations of estimates of $\sigma_{A_{\mathrm{sl}}}^{2}$ and $\sigma_{A_{v}}^{2}$ decreased substantially, e.g. from 50 to 200 offspring with 54 and 60%, respectively, whereas the standard deviation of estimates of $\sigma_{A_{\mathrm{int}}}^{2}$ decreased only slightly (−10%). When increasing the number of sire families from 50 to 200, standard deviations of estimates of all genetic parameters decreased substantially (from −52 to −58%). Based on these results, we conclude that designs with at least 100 sires with 100 offspring each are required in order to estimate variance components for macro- and micro-environmental sensitivities with low standard errors.

**Table 5:** Means and standard deviations across 100 replicates of estimated genetic parameters for different designs

		**Estimated parameters (SD)**						
**NS**	**NO**	$\sigma_{A_{\mathrm{int}}}^{2}$	$\sigma_{A_{\mathrm{sl}}}^{2}$	$\sigma_{A_{v}}^{2}$	$\rho_{A_{\mathrm{int}},A_{v}}$	$\rho_{A_{\mathrm{int}},A_{\mathrm{sl}}}$	$\rho_{A_{\mathrm{sl}},A_{v}}$	**Np**
100	20	0.301	0.063	0.120	−0.008	−0.004	0.083	51
		(0.057)	(0.038)	(0.113)	(0.304)	(0.216)	(0.416)	
100	50	0.309	0.056	0.099	−0.016	−0.013	0.025	8
		(0.051)	(0.020)	(0.060)	(0.279)	(0.165)	(0.376)	
100	100	0.315	0.054	0.107	−0.005	0.004	0.012	0
		(0.053)	(0.015)	(0.046)	(0.165)	(0.155)	(0.249)	
100	200	0.301	0.053	0.093	0.000	0.007	−0.013	0
		(0.046)	(0.009)	(0.024)	(0.135)	(0.116)	(0.168)	
50	100	0.312	0.053	0.107	0.015	0.002	0.036	5
		(0.079)	(0.018)	(0.064)	(0.295)	(0.199)	(0.320)	
200	100	0.301	0.053	0.099	0.009	−0.002	−0.009	0
		(0.033)	(0.009)	(0.028)	(0.142)	(0.108)	(0.163)	

NS = number of sires; NO = number of offspring per sire; $\sigma_{A_{\mathrm{int}}}^{2}$ = additive genetic variance of breeding value for intercept (true value = 0.3); $\sigma_{A_{\mathrm{sl}}}^{2}$ = additive genetic variance of breeding value for slope (= macro-environmental sensitivity; true value = 0.05); $\sigma_{A_{v}}^{2}\;$ = additive genetic variance for environmental variance (= micro-environmental sensitivity; true value = 0.10); $\rho_{A_{\mathrm{int}},A_{v}}$ = $\rho_{A_{\mathrm{int}},A_{\mathrm{sl}}}$ = $\rho_{A_{\mathrm{sl}},A_{v}}\;$ = 0; Np = number of replicates with covariance structures forced to be positive definite.

### Model selection

Using AIC to select the best fitting model, the true genetic model was selected as the best statistical model in at least 90% of the 100 replicates when the number of offspring per sire was 100 (Table 6). In only a few cases did the best statistical model differ from the true genetic model. However, when the number of offspring per sire was 50, the correct model was selected for 63% to 95% of the replicates. When both macro- and micro-environmental sensitivity existed and the number of offspring per sire was 50, the macro model was selected rather than the macro–micro model in 27% of the replicates. When only micro-environmental sensitivity existed and 50 offspring per sire, the power to select the correct model was reduced. In conclusion, the power to select the correct model was high in designs with at least 100 offspring per sire.

**Table 6:** The best model selected in 100 replicates according to Akaike’s information criterion and effect of the number of offspring for different true genetic models

**NO**	**True genetic model**	**True parameters**		**Nb of times model selected based on AIC**			
		$\sigma_{A_{v}}^{2}$	$\sigma_{A_{\mathrm{sl}}}^{2}$	**Macro–micro**	**Macro**	**Micro**	**Simple**
100	Macro–micro	0.1	0.05	99	1	0	0
	Macro	0	0.05	5	95	0	0
	Micro	0.1	0	3	1	94	2
	Simple	0	0	1	4	5	90
50	Macro–micro	0.1	0.05	63	27	8	2
	Macro	0	0.05	7	87	1	5
	Micro	0.1	0	4	1	71	24
	Simple	0	0	1	1	3	95

NO = number of offspring per sire, “Macro–micro” = model accounting for both macro- and micro-environmental sensitivities; “Macro” = model with only macro-environmental sensitivity; “Micro” = model with only micro-environmental sensitivity; “Simple” = model without macro- and micro environmental sensitivities and only a genetic effect for the phenotype; $\sigma_{A_{\mathrm{int}}}^{2}$ = additive genetic variance of breeding value for intercept (true value = 0.3); $\sigma_{A_{\mathrm{sl}}}^{2}$ = additive genetic variance of breeding value for slope (= macro-environmental sensitivity), $\sigma_{A_{v}}^{2}\;$ = additive genetic variance for environmental variance (= micro-environmental sensitivity), $\rho_{A_{\mathrm{int}},A_{v}}$ = $\rho_{A_{\mathrm{int}},A_{\mathrm{sl}}}$ = $\rho_{A_{\mathrm{sl}},A_{v}}$ = 0.

### Scenarios in which true genetic and statistical models differ

It is of interest to determine whether genetic parameters are biased if the wrong statistical model is applied. When either macro or micro-environmental sensitivity was simulated (Table 7), the variance-covariance structures were forced to be positive definite in many replicates, due to estimates of variances at the boundary. Estimates at the boundary were expected since the estimated genetic variance should be close to zero when the true genetic variance is zero. Forcing the genetic variance-covariance structures to be positive definite can bias estimates of parameters. When true macro-environmental sensitivity existed, some micro-environmental sensitivity was detected ( ${\hat{\sigma }}_{A_{v}}^{2}>0$) with the “micro” (scenario A) and the “macro–micro” (scenario B) models, indicating that part of the macro-environmental sensitivity was captured as micro-environmental sensitivity, but ${\hat{\sigma }}_{A_{v}}^{2}$ were small (0.010-0.018). When true micro-environmental sensitivity existed, no macro-environmental sensitivity was detected ( ${\hat{\sigma }}_{A_{\mathrm{sl}}}^{2}\approx 0$) with the “macro” (scenario C) and “macro–micro” (scenario D) models, but ${\hat{\sigma }}_{A_{v}}^{2}$ were biased downwards (between −16% to −28%), possibly because of the high number of genetic variance-covariance structures that were forced to be positive definite. When both macro and micro-environmental sensitivity existed, the “macro” (scenario E) and “micro” (scenario F) models gave unbiased estimates of either macro-environmental sensitivity or micro-environmental sensitivity and the genetic variance-covariance structure was forced to be positive definite for at maximum three replicates. Thus, the general conclusion is that a discrepancy between the true and statistical models does not lead to large biases in estimated genetic parameters and that contamination between estimates of the two types of environmental sensitivity is very limited.

**Table 7:** Means and standard deviations across 100 replicates of estimated genetic parameters when true and statistical models differ

**Scenario**	**True model**	**True parameters**		**Statistical model**	**Estimated parameters (SD)**			
		$\sigma_{A_{\mathrm{sl}}}^{2}$	$\sigma_{A_{v}}^{2}$		$\sigma_{A_{\mathrm{int}}}^{2}$	$\sigma_{A_{\mathrm{sl}}}^{2}$	$\sigma_{A_{v}}^{2}$	**Np**
A	Macro	0.025	0	Micro	0.303		0.010	51
					(0.045)		(0.020)	
	Macro	0.05	0	Micro	0.303		0.010	48
					(0.044)		(0.013)	
	Macro	0.1	0	Micro	0.308		0.018	37
					(0.050)		(0.019)	
B	Macro	0.025	0	Macro–micro	0.296	0.027	0.012	60
					(0.046)	(0.009)	(0.011)	
	Macro	0.05	0	Macro–micro	0.298	0.052	0.013	51
					(0.048)	(0.011)	(0.013)	
	Macro	0.1	0	Macro–micro	0.307	0.091	0.012	54
					(0.051)	(0.020)	(0.013)	
C	Micro	0	0.05	Macro	0.296	0.002		0
					(0.041)	(0.003)		
	Micro	0	0.1	Macro	0.292	0.003		0
					(0.039)	(0.004)		
	Micro	0	0.2	Macro	0.289	0.002		0
					(0.043)	(0.003)		
D	Micro	0	0.05	Macro–micro	0.303	0.003	0.053	71
					(0.050)	(0.003)	(0.024)	
	Micro	0	0.1	Macro–micro	0.300	0.003	0.084	60
					(0.047)	(0.003)	(0.034)	
	Micro	0	0.2	Macro–micro	0.304	0.002	0.143	66
					(0.049)	(0.003)	(0.058)	
E	Macro–micro	0.05	0.05	Macro	0.297	0.055		0
					(0.047)	(0.014)		
	Macro–micro	0.05	0.1	Macro	0.298	0.053		0
					(0.052)	(0.012)		
	Macro–micro	0.05	0.2	Macro	0.292	0.053		0
					(0.049)	(0.011)		
	Macro–micro	0.025	0.1	Macro	0.306	0.026		0
					(0.050)	(0.008)		
	Macro–micro	0.1	0.1	Macro	0.298	0.106		0
					(0.045)	(0.022)		
F	Macro–micro	0.05	0.05	Micro	0.299		0.050	3
					(0.051)		(0.031)	
	Macro–micro	0.05	0.1	Micro	0.297		0.095	1
					(0.047)		(0.042)	
	Macro–micro	0.05	0.2	Micro	0.304		0.191	0
					(0.045)		(0.070)	
	Macro–micro	0.025	0.1	Micro	0.299		0.105	0
					(0.049)		(0.043)	
	Macro–micro	0.1	0.1	Micro	0.298		0.103	1
					(0.045)		(0.045)	

See Table 2 for schematic overview of scenarios; “Macro–micro” = model accounting for both macro- and micro-environmental sensitivities; “Macro” = model with only macro-environmental sensitivity; “Micro” = model with only micro-environmental sensitivity; $\sigma_{A_{\mathrm{int}}}^{2}$ = additive genetic variance of breeding value for intercept (true value = 0.3); $\sigma_{A_{\mathrm{sl}}}^{2}$ = additive genetic variance of breeding value for slope (= macro-environmental sensitivity); $\sigma_{A_{v}}^{2}\;$ = additive genetic variance for environmental variance (= micro-environmental sensitivity), $\rho_{A_{\mathrm{int}},A_{v}}$ = $\rho_{A_{\mathrm{int}},A_{\mathrm{sl}}}$ = $\rho_{A_{\mathrm{sl}},A_{v}}\;$ = 0; Np = number of replicates with covariance structures forced to be positive definite.

### Application to milk yield in dairy cattle

The “macro–micro”, “macro”, “micro” and “simple” models were applied to 305-day first lactation milk yield data of Swedish Holsteins (mean = 8693 kg, standard deviation = 1652 kg, skew = 0.18, kurtosis = 0.28). “Macro”, “micro” and “macro–micro” models fitted significantly better than the “simple” model. The “micro” model was favoured by AIC (Table 8) and had the best fit. The genetic variance for micro-environmental sensitivity was substantial but lower than for most reported traits [20]: one genetic standard deviation changed micro-environmental sensitivity (= environmental variance) by 21%. The difference in AIC between the “macro–micro” and “micro” models was small and therefore it was interesting to examine the genetic parameters of the “macro–micro” model. The estimated genetic variance for macro-environmental sensitivity was small in comparison to the genetic variance in intercept. For instance, the estimate of the genetic correlation between environments that were −2 and 2 standard deviations from the overall mean was 0.92, indicating a small level of reranking of sires across the environmental gradient. Estimates of genetic correlations between intercept and macro- and micro-environmental sensitivities were 0.81 and 0.63, respectively, indicating that selection for higher milk yield increases both types of environmental sensitivity. The estimate of the genetic correlation between macro- and micro-environmental sensitivities was 0.76, indicating that they are genetically similar. Standard errors of parameter estimates were smaller than the standard deviations found for the simulations, due to the larger dataset i.e. more sires and more offspring per sire. Thus, macro- and micro-environmental sensitivities may exist for milk yield in dairy cattle and are positively correlated.

**Table 8:** Estimated genetic parameters for macro- and micro-environmental sensitivity of milk yield in dairy cattle

**Parameter**	**Macro–micro**		**Macro**		**Micro**		**Simple**	
	**Estimate**	**SE**	**Estimate**	**SE**	**Estimate**	**SE**	**Estimate**	**SE**
$\sigma_{A_{\mathrm{int}}}^{2}$	420 800	27960	420 400	28 004	416 800	27 696	416 000	27 692
$\sigma_{A_{\mathrm{sl}}}^{2}$	11 096	2288	11 116	2320				
$\sigma_{A_{v}}^{2}$	0.043	0.008			0.042	0.008		
$\rho_{A_{\mathrm{int}},A_{\mathrm{sl}}}$	0.808	0.062	0.812	0.063				
$\rho_{A_{\mathrm{int}},A_{v}}$	0.627	0.073			0.608	0.0751		
$\rho_{A_{\mathrm{sl}},A_{v}}$	0.765	0.098						
APHL	193 704		194 179		193 692		202 832	
AIC	193 722		194 191		193 704		202 840	

$\sigma_{A_{\mathrm{int}}}^{2}$ = additive genetic variance of breeding value for intercept; $\sigma_{A_{\mathrm{sl}}}^{2}$ = additive genetic variance of breeding value for slope (= macro-environmental sensitivity); $\sigma_{A_{v}}^{2}\;$ = additive genetic variance in environmental variance (= micro-environmental sensitivity); $\rho_{A_{\mathrm{int}},A_{v}}$ = genetic correlation between breeding value for intercept and environmental variance; $\rho_{A_{\mathrm{int}},A_{\mathrm{sl}}}$ = genetic correlation between breeding values of intercept and slope of reaction norm; $\rho_{A_{\mathrm{sl}},A_{v}}$ = genetic correlation between breeding values of slope and environmental variance; APHL = adjusted profile h-likelihood; AIC = Akaike’s information criterion; “Macro–micro” = model accounting for both macro- and micro-environmental sensitivities; “Macro” = model with only macro-environmental sensitivity; “Micro” = model with only micro-environmental sensitivity; “Simple” = model without macro- and micro environmental sensitivities and only a genetic effect for the phenotype; SE = approximate standard error obtained with ASReml.

## Discussion

### Model and design

In this study, we developed a model to estimate genetic variances for macro- and micro-environmental sensitivities. The model is an extension of the DHGLM as presented by Rönnegård et al. [19]. Here, we combined a linear reaction norm model to estimate genetic variance for macro-environmental sensitivity with the DHGLM to estimate genetic variance for micro-environmental sensitivity. The animal model in Equation (2) was adapted to a sire model because the animal model produced highly biased estimated variance components because of the high dependence of the estimated breeding values and residuals on the variance ratio used in the mixed model equations, which would differ for each animal. Felleki et al. [31] also reported the presence of bias in variance components with few repeated observations per animal but the bias decreased as the number of repeated observations per animal increased. Furthermore, an animal model with heterogeneous residual variance (DHGLM implementation) gave a poorer adjusted profile h-likelihood than an animal model without homogenous residual variance, which indicates that the former did not produce a better fit than the latter in a scenario that included both macro and micro-environmental sensitivities. Therefore, we decided to use a sire model implementation, because it is more robust than the animal model implementation with DHGLM when animals only have a single observation. Furthermore, sire models are commonly used for reaction norm models, because of their substantially lower computational burden compared to animal models and genetic information about environmental sensitivity typically comes from paternal half-sibs that perform in different environments. As far as we know, this is the first time that a model to estimate genetic variance in macro- and micro-environmental sensitivities suitable for outbred animal populations is presented.

Monte Carlo simulation was used to evaluate bias and precision of estimated genetic parameters. Genetic parameters were unbiased in most situations. The precision was not very high, especially of estimates for $\sigma_{A_{v}}^{2}$, as indicated by the high standard deviation of estimates across replicates, particularly in designs with small families. Designs with at least 100 sire families, each with at least 100 offspring, are required to have sufficient precision. Presence of fixed effects, such as contemporary group effects, would increase the required number of sire families and the number of offspring per sire family. These results are in agreement with standard error and power calculations reported by Hill [39], Mulder [40] and Hill and Mulder [20] with respect to estimation of genetic variance for micro-environmental sensitivity or for environmental variance. For instance, Hill and Mulder [20] derived that the optimal family size to estimate genetic variance for environmental variance with a family design is approximately 2/*γ*^2^, where *γ*^2^ is the square of the coefficient of variation of the within-family variance. Thus, the optimal family size for half-sibs is 137 when $\sigma_{A_{v}}^{2}=0.10$. The only study providing Monte Carlo results for the DHGLM to estimate genetic variance for micro-environmental sensitivity is by Rönnegård et al. [19]. They considered a design with clones, which is more powerful and leads to lower standard deviations of estimates than those in our study.

With respect to macro-environmental sensitivity, the magnitude of the standard deviations of the $\sigma_{A_{\mathrm{sl}}}^{2}$ estimates across replicates was similar to that reported by Calus et al. [36], but larger than those reported by Lillehammer et al. [41], which is explained by the fact that the latter authors simulated more sire families, i.e., 1000 sire families with 100 offspring each. In general, the number of required offspring per family is lower for macro-environmental sensitivity than for micro-environmental sensitivity, as indicated by the lower standard deviations of estimates of genetic variance for the former. This is in agreement with a previous study by Mulder [40], which showed that the power to detect G x E interactions between two environments is greater than the power to detect genetic variance for environmental variance or micro-environmental sensitivity. In the present study, we assumed that the environmental parameter *x* used for the reaction norm was known without error. This will be true in some cases, e.g. when using temperature or rainfall or other herd characteristics [42]. In other cases, an estimated herd mean is used as the environmental parameter, which is estimated from the data [7,8,33]. Calus et al. [36] showed that genetic variance in macro-environmental sensitivity ( $\sigma_{A_{\mathrm{sl}}}^{2}$) was severely underestimated when the environmental parameter was estimated from the data. This may have also led to the genetic variance in macro-environmental sensitivity to be underestimated in our application to milk yield. Su et al. [43] reported that a Bayesian approach that estimates simultaneously the herd mean and the reaction norm parameters leads to unbiased estimates of $\sigma_{A_{\mathrm{sl}}}^{2}$.

Lillehammer et al. [41] showed that sire models gave upward biased estimates of $\sigma_{A_{\mathrm{sl}}}^{2}$ when heterogeneity of residual variance was ignored, because the unexplained genetic variance in the reaction norm parameters becomes part of the residual variance when using a sire model. In our study, the bias in estimates of $\sigma_{A_{\mathrm{sl}}}^{2}$ was smaller than in Lillehammer et al. [41]. Lillehammer et al. [41] proposed including a dummy animal effect in the model to account for the residual three-quarters of the genetic variance that is not accounted for by the sire effect. This solution was also tested in our model but gave severely biased variance components because of the high dependency of estimated breeding values and residuals on variance ratios that were used in the mixed model equations, which differ by animal when considering heterogeneity of residual variance.

The algorithm developed in this study allowed estimating genetic correlations between the different genetic effects. Standard deviations of estimated genetic correlations were large, especially those of the genetic correlation between macro- and micro-environmental sensitivities ( $\rho_{A_{\mathrm{sl}},A_{v}}$). This large magnitude of the standard deviation of estimates of the genetic correlation was expected considering that the genetic correlation between macro- and micro-environmental sensitivities is mainly based on paternal half-sib information and that both traits have low heritability. Using the equation in Robertson [44] and assuming a heritability of 0.05 for both traits, the standard error is approximately 0.26 when the true genetic correlation between the traits is zero, which is close to the value found here, i.e. 0.25 (Table 4). To increase the precision of estimates of the genetic correlation between macro- and micro-environmental sensitivities, designs with a larger number of families and larger family sizes are required. The application to milk yield data in dairy cattle shows that it should be possible to estimate genetic correlations with standard errors between 0.06 and 0.10, since in most countries datasets with at least 100 bulls each with 100 daughters are easily obtained. Full-sib families or clones would also reduce standard errors of estimates of genetic correlations in comparison to half-sibs.

Here, we showed that the adjusted profile h-likelihood (APHL) can be approximated from REML-output and used in combination with AIC to provide an efficient model selection tool. In addition, we showed that biases in genetic parameters were relatively small when statistical and true models differed. Both results are re-assuring that these models can discriminate between macro- and micro-environmental sensitivities and that the true model of environmental sensitivity can be elucidated using AIC. We also found that the Bayesian information criterion (BIC) was too conservative and favoured the simpler model too often (results not shown). AIC has the advantage that it can be used independent of the order of the fitted models, whereas the likelihood ratio test requires a hierarchical structure such as in a forward selection scheme [45].

### Improving biological understanding of environmental sensitivity

The proposed model can contribute to better understand the genetic architecture of environmental sensitivity, e.g. whether macro- and micro-environmental sensitivities are genetically related. Most studies in plants and laboratory species indicate that macro- and micro-environmental sensitivities are weakly correlated [22-24,26-28]. This seems to indicate that selection on one type of environmental sensitivity will hardly affect the other. The first application of the model on milk yield data in dairy cattle, revealed a high genetic correlation between macro- and micro-environmental sensitivities (0.76), suggesting that selection on one type of environmental sensitivity will also affect the other in the same direction. Generally, little is known about these relationships in livestock. Knowledge about these genetic correlations could be used to optimize selection strategies for environmental sensitivity.

In this study, we assumed a linear reaction norm model, but reaction norms can also be non-linear [8]. The model presented here can easily be extended to higher-order polynomials. Furthermore, the genetic basis of micro-environmental sensitivity may not be the same along an environmental gradient and the model for residual variance or micro-environmental sensitivity in Equation (3) could be extended to contain a reaction norm with a known environmental gradient. For instance, in stressful environments, there might be more genetic variance for micro-environmental sensitivity than in less stressful environments. Furthermore, G × E interactions often exist between categorical environments and are often analysed with character state or multivariate models [2]. Character state models do not explicitly estimate breeding values for macro-environmental sensitivity, but these breeding values could be back-calculated by using covariance functions when the environmental parameter responsible for G × E interactions is identified because reaction norm models and character state models are interchangeable [2]. In the case of two environments, a reaction norm model with a dummy environmental parameter with values 0 and 1 would yield results that are identical to a bivariate character state model. Multivariate versions of the DHGLM or reaction norm models with dummy environmental variables could be used to simultaneously investigate macro- and micro-environmental sensitivities when environments are discrete.

### Application to breeding

Taking macro- and micro-environmental sensitivities into consideration is highly relevant in animal breeding. Due to the high level of globalisation in animal breeding programs, it is necessary to breed animals that can perform well in a wide range of environments. Therefore, it may be important to select animals that have limited environmental sensitivity, especially for environments with a higher risk of environmental disturbances. Reduction in environmental sensitivity increases the predictability of performance and reduces risk for farmers [46]. Furthermore, reduction in micro-environmental sensitivity will increase the uniformity of animal products [47], which is a general goal. In plant breeding, application of a model for macro- and micro-environmental sensitivities is also highly relevant since G × E interactions are generally very strong and uniformity of crops is very important. Recent papers by Ordas et al. [48], Makumburage and Stapleton [49] and Kliebenstein [50] show that there is an interest for increased uniformity in plants. Economic values could be derived for micro-environmental sensitivity [47]. The economic value of macro-environmental sensitivity can be determined as a function of the importance of environments along the environmental gradient. Based on Mulder et al. [3,47], progeny testing schemes are more efficient than sib testing schemes to reduce micro-environmental sensitivity since it behaves as a trait with a small heritability. Genomic selection could be an alternative selection strategy with sufficient accuracy and shorter generation intervals.

## Conclusions

In this study, a model was developed to estimate genetic parameters of macro- and micro-environmental sensitivities, combining a reaction norm model with a double hierarchical generalized linear model within a REML framework. Simulations showed that the genetic parameters obtained were mostly unbiased, but designs with at least 100 sires, each with 100 half-sib offspring, were required to estimate genetic parameters with sufficient precision. Using AIC, the true genetic model was selected as the best statistical model in at least 90% of replicates when the number of offspring per sire was 100. Application of the model to milk yield data in dairy cattle showed that both types of environmental sensitivity existed. Our model and AIC based on h-likelihood can be used to increase our understanding of the genetic control of environmental sensitivity in livestock populations but more research is needed to test the model in a wider range of situations.

## Appendix

### DHGLM algorithm for a sire model

The original DHGLM algorithm of Rönnegård et al. [19] was developed for an animal model. Here we describe the estimation algorithm for the sire model used in the current paper, including a few adjustments of the algorithm in Rönnegård et al. [19] to correct for the fact that the residual variance in a sire model (without permanent environmental effects, e.g. with animals with a single observation) contains three quarters of the additive genetic variance in addition to the environmental variance. The adjustments in the algorithm are as follows: adjustment of the squared residuals (**y**_v_) accounting for the fact that the residual variance in a sire model includes three quarters of the additive genetic variance, use of average residual variance ${\hat{\sigma }}_{e_{s}}^{2}$ to calculate **ψ**_**s**_ (instead of predicted individual values), and computations of the diagonal weight matrices **W**_**s**_ and **W**_**sv**_. These adjustments resulted in a computationally robust algorithm with small or no bias (as presented in Results).

To compute the linearized response **ψ**_**s**_ for the bivariate sire model in Equation (3), first we calculate $y_{v_{i}}$ as:

(6) $$y_{v_{i}}=\frac{{\hat{e}}_{s_{i}}^{2}}{\left(1-h_{i}\right)}\times \frac{\bar{{\hat{\sigma }}_{e_{s}}^{2}}}{\bar{{\hat{\sigma }}_{e_{a}}^{2}}}.$$

This is equivalent to the calculations for the response *y*_v_ in [19], except for the multiplication by the ratio of the average estimated residual variance in a sire model ( $\bar{{\hat{\sigma }}_{e_{s}}^{2}}=\sigma_{\in_{s}}^{2}/\bar{w_{s}}$, where $\bar{w_{s}}=\mathrm{tr}\left(W_{s}\right)/n$and *n* is the total number of records) and the average residual variance in an animal model, which is calculated as $\bar{{\hat{\sigma }}_{e_{a}}^{2}}=\bar{{\hat{\sigma }}_{e_{s}}^{2}}-\frac{3}{4}{\hat{\sigma }}_{A_{\mathrm{int}}}^{2}$, ( σ^Aint2=4σ^sint2). Because we use a log link function, $y_{v_{i}}$ is linearized as:

(7) $$\psi_{s}{}_{{}_{i}}=\log\left(\bar{{\hat{\sigma }}_{e_{s}}^{2}}\right)+\frac{y_{v}{}_{{}_{i}}-\bar{{\hat{\sigma }}_{e_{s}}^{2}}}{\bar{{\hat{\sigma }}_{e_{s}}^{2}}}.$$

The diagonals of **W**_s_ are the reciprocals of ${\hat{\psi }}_{s}$ (i.e. $W_{s}{}_{{}_{i}}=1/{\hat{\psi }}_{s_{i}}$), which is the vector of predicted residual variances for each observation based on the previous iteration and is calculated as:

(8) $${\hat{\psi }}_{s_{i}}=\exp\left(\log\left(\bar{{\hat{\sigma }}_{e_{s}}^{2}}-\frac{3}{4}{\hat{\sigma }}_{A_{\mathrm{int}}}^{2}\right)+s_{v}{}_{{}_{i}}\right)+\frac{3}{4}\;{\hat{\sigma }}_{A_{\mathrm{int}}}^{2}.$$

The sire effects for environmental variance $s_{v_{i}}$ only affect the part of the residual variance which is truly environmental variance and therefore three-quarters of the additive genetic variance is subtracted in the multiplicative part of Equation (8). The diagonals of **W**_**sv**_ are the reciprocals of the residual variance of **ψ**_**s**_ and were calculated as:

(9) $$W_{sv_{i}}=\frac{1}{2}{\left(1-h_{i}\right)}^{2}{\left(\frac{\bar{{\hat{\sigma }}_{e_{a}}^{2}}}{\bar{{\hat{\sigma }}_{e_{s}}^{2}}}\right)}^{2}.$$

This is motivated by the fact that by combining Equations (6) and (7), and by assuming that the estimated residuals are close to the true ones, we have:

(10) $$\begin{array}{l} \mathrm{var}\left(\psi_{s}{}_{i}\right)=\mathrm{var}\left(\frac{e_{s_{i}}^{2}}{\left(1-h_{i}\right)}\times \frac{\bar{{\hat{\sigma }}_{e_{s}}^{2}}}{\bar{{\hat{\sigma }}_{e_{a}}^{2}}}\times \frac{1}{\bar{{\hat{\sigma }}_{e_{s}}^{2}}}\right)\\ \;=\mathrm{var}\left(\frac{e_{s_{i}}^{2}}{\left(1-h_{i}\right)}\times \frac{1}{\bar{{\hat{\sigma }}_{e_{a}}^{2}}}\right)=2{\left(1-h_{i}\right)}^{-2}{\left(\frac{\bar{{\hat{\sigma }}_{e_{s}}^{2}}}{\bar{{\hat{\sigma }}_{e_{a}}^{2}}}\right)}^{2}. \end{array}$$

since the true residuals are assumed normal and the squared true residuals are therefore Gamma distributed with a variance of $2{\left({\hat{\sigma }}_{e_{s}}^{2}\right)}^{2}$.

The algorithm can be summarized as:

1. Run model on **y** in Equation (3) with homogeneous residual variance.

2. Calculate **ψ**_**s**_, **W**_**s**_, **W**_**sv**_, where $W_{s}=\mathrm{diag}\left({\hat{\sigma }}_{e}^{2}\right)$ in iteration 1.

3. Run bivariate model in Equation (3).

4. Update **ψ**_**s**_, **W**_**s**_, **W**_**sv**_

5. Iterate steps 3 till 4 until convergence

Approximation of the adjusted profile h-likelihood

In our paper, the model selection was based on an approximation of the adjusted profile h-likelihood (APHL), which is defined as [51]:

(11) $$\mathrm{APHL}=\left(2h-\log\left(\det\left(H\right)\right)\right){|}_{\tau =\hat{\tau }},$$

where **H** is the Hessian of the h-likelihood and ***τ*** is the vector of all fixed and random effects both in the mean and variance parts of the model. For the model in Equation (3), minus two times the h-likelihood (− 2*h*) is:

(12) −2h=−2ly|sint,ssl,sv+lsint,ssl,sv=∑i=1nlogσ^ei2+e^i2σ^ei2+log(detG˜)+s˜'G˜−1s˜,

where *l* is log-likelihood, ${\hat{\sigma }}_{e_{i}}^{2}$ ( ${\hat{\sigma }}_{e_{i}}^{2}={\hat{\sigma }}_{\in_{s}}^{2}/w_{s_{i}}$, with $w_{s_{i}}$ being the *i*^*th*^ diagonal of **W**_**s**_) is the residual variance of observation *i*, ${\hat{e}}_{i}^{2}$ is the estimated squared residual of observation *i*, $\tilde{G}$ is the covariance matrix of all random effects ( $\tilde{G}=\frac{1}{4}G⊗A$), **s** is a vector of all random sire effects ( s˜'=sintsslsv) and $w_{s_{i}}$ is the weight for the mean model for observation *i*. The minus two log REML likelihood (*logL*) from the bivariate model in Equation (3) is:

(13) −2logL=∑i=1n+klogσ^ei2+e^i2σ^ei2+logdetG˜+s˜'G˜−1s˜+logdetC,

where the first *n* residual variances come from the first part of the bivariate model (**y**) and the next *k* residual variances come from the second part of the bivariate model (**ψ**_**s**_) (*k* = *n* = number of records), and **C** is the Hessian of the bivariate model (i.e. left-hand-side of the mixed model equations). Because log (det(**C**)) is a reasonable approximation of log (det (**H**)) [31], we can approximate *APHL* as given in Equation (4) in the main text:

(14) $$\mathrm{APHL}=-2\mathrm{logL}-\sum w_{sv_{i}}{\left({\hat{\sigma }}_{\in_{\mathrm{sv}}}^{2}\right)}^{-1}-\sum \ln\left({\hat{\sigma }}_{\in_{\mathrm{sv}}}^{2}\right)/w_{sv_{i}}$$

Thus, the REML likelihood for the bivariate model is corrected for the fact that the squared residuals are used as “observations” in the bivariate model. Note that equation (14) can also be used for animal models by replacing the elements $w_{sv_{i}}$ and $\sigma_{\varepsilon_{\mathrm{sv}}}^{2}$ with the corresponding elements of Equation (2) or for models with more or fewer random effects in the mean and variance model.

## Competing interests

The authors declare that they have no competing interests.

## Authors’ contributions

All authors were involved in designing the study and revising the manuscript. HAM developed the model, performed the simulations, analysed the data and drafted the manuscript. LR developed the adjusted profile h-likelihood. All authors read and approved the final manuscript.
